# Reply: Hereditary myopathy with early respiratory failure is caused by mutations in the titin FN3 119 domain

**DOI:** 10.1093/brain/awu034

**Published:** 2014-02-27

**Authors:** Gerald Pfeffer, Patrick F. Chinnery

**Affiliations:** Institute of Genetic Medicine, Newcastle University; and Department of Neurology, Royal Victoria Infirmary, Newcastle, NE1 3BZ, UK

Sir, The response by Lange *et al.* (2014) provides important confirmatory information. The coincidence of the FN 119 domain mutation (p.P30091L) with the kinase domain variant (p.R32450W or R279W) in all patients previously reported by Lange *et al.* (2005) confirms speculation (Hedberg *et al.*, 2013; Pfeffer *et al.*, 2013) that the HMERF in the original family was primarily a result of the p.P30091L FN 119 mutation, and not to the kinase domain variant (p.R32450W or R279W). The kinase domain variant (p.R32450W or R279W) was originally reported to be the cause of HMERF by Lange *et al.* (2005), but has subsequently been reported in healthy control subjects. On the other hand, the p.P30091L FN 119 mutation has been reported repeatedly and exclusively in HMERF families.

Lange *et al.* (2014) suggest that we contradict ourselves regarding the pathogenicity of the p.P30091L variant, citing out of context (and out of chronological order) our report in which we considered p.P30091L as a possible neutral variant (Pfeffer *et al.*, 2014). However, we had also written that the variant may be pathogenic with variable penetrance and/or expressivity. At that time, it was the first published report of HMERF with this mutation, so we could not reach a final conclusion with confidence. Since then, another study confirmed the association of the p.P30091L mutation with HMERF (Palmio *et al.*, 2013), and in retrospect, a patient with this mutation and ‘myopathy with cytoplasmic aggregates’ (Vasli *et al.*, 2012) could also have HMERF. Lange *et al.* (2014) now report that their HMERF patients originally reported in 2005 have this same mutation. Although we had previously been uncertain regarding the pathogenicity of the p.P30091L variant, current evidence repeatedly links this mutation with HMERF (Vasli *et al.*, 2012; Hedberg *et al.*, 2013; Palmio *et al.*, 2013; Pfeffer *et al.*, 2014; Lange *et al.*, 2014).

The very interesting question is the reason for the more severe disease expressivity and complete penetrance in the patients reported (Edstrom *et al.*, 1990; Lange *et al.*, 2005), compared with other patients who have the p.P30091L mutation (Palmio *et al.*, 2013; Pfeffer *et al.*, 2014). Lange *et al.* (2014) hypothesize that the kinase domain variant is the cause of the more severe phenotype in their patients. For the time being, there is no additional evidence to support this hypothesis. As indirect evidence, Lange *et al.* (2014) cite the importance of recessive kinase domain mutations in a recent publication (Chauveau *et al.*, 2013), although the principal clinical finding in most of these patients was cardiomyopathy, which does not occur in HMERF patients, and the patients have a different mode of inheritance (HMERF is autosomal dominant).

We attempted to find evidence for a modifier effect for kinase variants by screening a HMERF population of 33 patients, but our findings did not support this hypothesis (Pfeffer *et al.*, 2013). At present we can only reach the following conclusions with any confidence: (i) HMERF is caused by mutations in the FN119 domain of *TTN* (Ohlsson *et al.*, 2012; Pfeffer *et al.*, 2012, 2013*a*; Vasli *et al.*, 2012; Hedberg *et al.*, 2013; Izumi *et al.*, 2013; Palmio *et al.*, 2013; Toro *et al.*, 2013; Lange *et al.*, 2014); (ii) one particular mutation in the FN119 domain, p.P30091L, has variable expressivity and penetrance (Palmio *et al.*, 2013; Pfeffer *et al.*, 2014; Lange *et al.*, 2014), and the inheritance pattern may be dominant (Lange *et al.*, 2005; Pfeffer *et al.*, 2014) or may be recessive (Palmio *et al.*, 2013); (iii) in two screening studies of patients with undiagnosed myofibrillar myopathy, sequencing of 172 families identified nine patients with FN119 domain mutations, but none with kinase domain mutations (Pfeffer *et al.*, 2014; Toro *et al.*, 2013); (iv) the kinase variant of interest (R32450W/R279W) is reported in controls (it is listed in dbSNP as rs140319117 with an allele frequency of 0.2%, and we have also recently reported a healthy control with this variant) (Pfeffer *et al.*, 2013). This kinase variant does not appear to be capable of causing HMERF; and (v) R32450W/R279W has only been associated with HMERF in the original report by Lange *et al.* (2005). In this family, a common haplotype contains both a FN119 domain mutation and the kinase domain variant (Lange *et al.*, 2014). Given the more severe phenotype in these patients, it is possible (but not proven) that the kinase domain mutation may be a modifier of the condition caused by the mutation in the FN119 domain. Other possible explanations include a different monogenic factor on the same haplotype, complex genetic factors, environmental factors, or a combination of these. Further evidence is required to substantiate the hypothesis proposed by Lange *et al.* (2014), that R32450W/R279W modifies the phenotype of HMERF.
